# Computational analysis of the structural-functional dynamics of a Co-receptor proteoglycan

**DOI:** 10.3389/fmolb.2025.1549177

**Published:** 2025-03-25

**Authors:** Francesco Tavanti, Giorgia Brancolini, Roberto Perris

**Affiliations:** ^1^ COMT – Centre for Molecular and Translational Oncology, University of Parma, Parma, Italy; ^2^ Department of Chemical and Life Sciences and Environmental Sustainability, University of Parma, Parma, Italy; ^3^ Center S3, CNR Institute of Nanoscience – CNR-NANO, Modena, Italy

**Keywords:** proteoglycans, molecular dynamics, computer simulations, co-receptor, growth factor

## Abstract

Nerve-Glial Antigen 2/Chondroitin Sulphate Proteoglycan 4 (NG2/CSPG4) is the largest membrane-intercalated cell surface component of the human proteome known to date. NG2/CSPG4 is endowed with the capability of engaging a myriad of molecular interactions and exert co-receptor functions, of which primary ones are sequestering of growth factors and the anchoring of cells to the extracellular matrix. However, the nature of the interactive dynamics of the proteoglycan remains veiled because of its conspicuous size and structural complexity. By leveraging on a multi-scale *in silico* approach, we have pioneered a comprehensive computational analysis of the structural-functional traits of the NG2/CSPG4 ectodomain. The modelling highlights an intricate assembly of β-sheet motifs linked together by flexible loops. Furthermore, our *in silico* predictions highlight that the previously delineated D1 domain may consistently remain more accessible for molecular interplays with respect to the D2 and D3 domains. Based on these findings, we have simulated the structural mechanism through the proteoglycan may serve as a co-receptor for growth factor FGF-2, showing that NG2/CSPG4 bends towards the receptor FGFR-1 for this growth factor and confirming the previously hypothesized trimeric complex formation promoted by FGF-2 dimers bridging the FGFR-1-proteoglycan interaction. The Chondroitin Sulphate Proteoglycan 4 is a large multi-domain transmembrane protein involved in several biological processes including pathological conditions. Despite its importance, it has never been studied at the atomistic level due to its large size. Here, we employed a multi-scale computer simulations approach to study its three-dimensional structure, its movements and co-receptor properties, showing that it can serve as mediator in the growth factor signaling process.

## 1 Introduction

NG2/CSPG4 proteoglycan is a very large and complex transmembrane macromolecule exposed on the surface of vertebrate cells. Uniqueness and multivalent traits of the NG2/CSPG4 are believed to be accounted by its extended core protein encompassing 2,325 residues and the wide spectrum of glycoforms generated by post-translational modifications. Incipient ultrastructural analyses (Nishiyama et al., 1991; Tillet et al., 1997) and a revisited amino acid sequence evaluation of the ectodomain of the human NG2/CSPG4 homologue, which spans 2,221 residues, has identified three distinct subdomains: a globular *N-*terminal domain (D1), a flexible rod-like central segment (D2), and a *C-*terminal domain (D3) assuming a globular conformation (Stallcup, 2002; Tamburini et al., 2019). The D3 domain contains 4 phylogenetically conserved Ca^2+^-binding β-sheet-type cadherin-type repeats (Staub et al., 2002) connected to each by a flexible loop, whereas the membrane proximal portion of the D3 domain encompasses two juxtaposed proteolytic cleavage sites.

**FIGURE 1:**
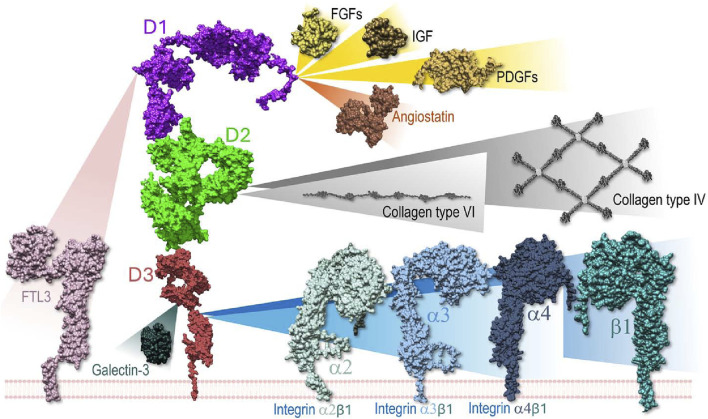
Schematic overview of the known molecular interactions engaged by the ectodomain of NG2/CSPG4 on the cell surface and in the extracellular space. In several instances the domains of the proteoglycan implicated in the interaction have been identified through the use of recombinant fragments, while the discrete amino acid residues involved in the bindings have not yet been disclosed. The same applies to the ligand molecules for which only in a few cases it has been possible to narrow down the NG2/CSPG4 interaction sites at a putative domain level. Through binding to collagens, the proteoglycan contributes to the stabilization of the cells’ attachment and directional movement on organized ECM structures. This integrin co-receptor function is complemented by a proposed modulatory activity exerted on selected (collagen/fibronectin-binding) integrins. However, the precise dynamics of this activity remains to be better defined. In the context of growth factor signaling, NG2/CSPG4 has been shown to act as a docking receptor, while the influence of the proteoglycan on the receptor activation remains to be explored more in detail. Sequestering of galectin-3 (and possibly galectin-9) is proposed to contribute to the proteoglycan’s involvement in cell-cell adhesion phenomena.

Despite its thoroughly documented impact on a plethora of biological processes (Figure 1), and the wide importance given to NG2/CSPG4 as a disease marker and therapeutic target (Garusi et al., 2012; Nicolosi et al., 2015; Cengiz et al., 2017; Ilieva et al., 2017; Rolih et al., 2017; Tamburini et al., 2019; Zhang et al., 2022), there is still scanty knowledge about the molecular interactions that NG2/CSPG4 engages on the cell surface and what the structural bases for these interactions may be. NG2/CSPG4 can mediate the interaction between membrane proteins, such as receptors and their ligands, mostly growth factors; bind several extracellular matrix (ECM) constituents and modulate the activity of integrin receptors; and link to molecules mediating the interaction of cells with their microenvironment.

Amongst the reproducibly documented molecular interactions of NG2/CSPG4, asserted by experimental means, are those occurring in a glycosaminoglycan-independent manner between the proteoglycan, growth factors (Nishiyama et al., 1996; Goretzki et al., 1999; Grako et al., 1999; Stallcup, 2002; Cattaruzza et al., 2013; Zhen et al., 2018), and their cognate receptors. Most recently, direct binding of NG2/CSPG4 to FTL3 has been disclosed on malignant hematopoietic cells (Lopez-Millan et al., 2024), while our unpublished observations suggest that the proteoglycan may also bind IGFs (*manuscript in preparation*). Thus, given the heterogeneity of the interactions and the intrinsic complexity of the proteoglycan, it may be deduced that its topographical arrangement on the cell membrane, alongside its three-dimensional structural dynamics, may dictate its multivalent functionality. However, only few attempts have been made to characterize the secondary and tertiary structure of the extracellular portion of the NG2/CSPG4, as well as define more in detail the modes of its molecular interplays. Next-generation technologies including advanced super resolution microscopy, cryo-EM and enhanced NMR approaches are progressively affording more detailed information about the mechanics of these molecular interactions, but these experimental methods have not yet reached the atomistic level of resolution. Meanwhile, software tools for computational *in silico* modelling have dramatically improved with the advent of artificial intelligence and machine learning methods, as well as algorithms and can now provide more accurate information on protein structural traits and predict more reliably finer molecular dynamics (Abramson et al., 2024). Notably, computational approaches offer the possibility to overcome experimental limitations, particularly those encountered with excessively large proteins (Wong et al., 2022).

There are three experimental entries to be found in the PDB; PDB IDs: 7ML7 (Chen et al., 2021), 7N8X and 7N9Y (Jiang et al., 2022) for NG2/CSPG4 which are reporting information on a short sequence of amino acids stretching from position 411 to 550 (Chen et al., 2021; Jiang et al., 2022). By contrast, no previous computational study is publicly available relative to the definition of the structural traits and interaction dynamics of the extracellular domain of the proteoglycan. Membrane intercalation *per se*, lateral mobility and micro-motion dictated by internal plasma membrane modulations are predicted to influence significantly the structural configuration of NG2/CSPG4 and thereby to impact on its putative biological functions (Ughrin et al., 2003; Tan et al., 2005). Previous computational analyses of an analogous proteoglycan, CD44, have merely focused on experimentally delineated subdomains with discrete putative functions (Nguyen et al., 2016; Lintuluoto et al., 2021), while neglecting other regions and possible interactions with the extracellular face of the membrane that could potentially modulate the overall dynamics of such cell surface protein.

We report here the first computational modeling of the full-length extracellular portion of the NG2/CSPG4 core protein and the predicted dynamics governing the molecular interactions that this region of the proteoglycan may promote. Our *in silico* analyses specifically address the challenges posed by the size and complexity of the proteoglycan and aim at offering new insights into its structural-functional properties that may be instrumental in gaining a better understanding of its biological role.

## 2 Methods

### 2.1 Atomistic molecular dynamics

The sequence of NG2/CSPG4 has been employed to reconstruct the three-dimensional structure using the AlpahFold3 webserver (Abramson et al., 2024). The sequence comprises 2,322 amino acids of which the first 2,221 are located in the extracellular matrix, residue from 2,222 to 2,246 form the transmembrane domain and residues from 2,247 to 2,322 form the cytoplasmatic domain. The best model obtained from AlphaFold3 (Abramson et al., 2024), i.e., the structure, has been employed as starting point for the following simulation procedure. The first 2,250 amino acids comprising the extracellular domain, the transmembrane domain and few amino acids of the cytoplasmatic domain have been used in this work. The CharmmGUI (Jo et al., 2008; Lee et al., 2019) webserver has been employed to insert the transmembrane helix into a DPPC membrane of size 29.854 nm × 29.854 nm comprising 3,320 lipid molecules packed into a double layer. The DPPC bilayer has been employed due to the presence of well-optimized potentials both for the CHARMM and Martini models. Moreover, its computational cost is lower with respect to longer lipid bilayer or complex mixtures. This is an important factor to take into account due to the large number of atoms involved in the system. For the simulation with protein free in solvent the box has size 25.74 × 27.11 × 40.66 nm for a total number of atoms of 2′810′270. For the simulation with protein and membrane, an orthorhombic box with height of 41.86 nm has been built using Gromacs 2023.3 (Abraham et al., 2015) by placing the protein-membrane complex into it, while maintaining the protein into the center. The box has been filled with TIP3P(MacKerell et al., 1998) water and ions and counterions have been added to mimic the physiological concentration of 0.15 mol/L and keeping the system neutral obtaining a total of 3′754′099 atoms. The system has been parameterized using the Charmm36 m force field (Huang et al., 2017) and the modified TIP3P water. Before the production run, an energy minimization has been performed until the forces reached a value lower than 1,000.0 kJ/mol/nm followed by an NVT equilibration at 310 K for 100 ps with a timestep of 2 fs and using the modified Berendsen thermostat with a coupling time of 0.1 ps. Then, an NPT equilibration has been performed for additionally 100 ps using the Parrinello-Rahman barostat with a coupling time of 2 ps and a semi-isotropic coupling to take into account the different compressibility along the XY plane for the membra ne and the Z direction for the protein and solvent. The production run has been performed for 200ns in the NPT ensemble using the Gromacs2023 package (Abraham et al., 2015). Two independent simulations, e.g., replicas, with different initial velocities and forces, have been conducted to enhance the sampling of the simulations. Furthermore, one of the replicas has been extended to 500 ns in order to observe possible changes at longer timescales.

#### 2.1.1 Coarse-grained molecular dynamics

The Martini2.2 Force Field (de Jong et al., 2013), which showed a good performance for glycoproteins dynamics (Chakraborty et al., 2021) has been employed to simulate the structure of the NG2/CSPG4 ectodomain linked to the membrane for long timescales. The mapping from atomistic to coarse-grained has been done using the Martinize2 package v2.6 (Monticelli et al., 2008; de Jong et al., 2013). The Martinize2 parameters for building the model are the following.

• Automatic choice of disulfide bonds (we check the consistency of this choice during the analysis)
• An elastic force with constant of 500 kJ/mol/nm^2^ was set and applied in the range 0.5–1.0 nm of distance
• The -ss flag has been employed in order to give as input the initial secondary structure of the protein obtained from AlphaFold model

In the SI we report the complete command of Martinize2 to build the model of the protein.

Simulations protocol is similar to the atomistic one reported above, with the exception that the timestep is set to 20fs and the production run is 5.5 µs long. Using the CG model, we performed two distinct replicas of the simulation changing in the initial configuration the atom velocities in a random way to have sample more different configurations. The simulation of the free protein in solvent has a box of 397.24 × 397.24 × 397.24 nm for a total of 530′609 beads, while the protein-membrane box has size of 297.24 × 297.24 × 384.69 nm for a total of 315′114 beads.

### 2.2 Computational analysis

The Root Mean Square Deviation (RMSD), the Root Mean Square Fluctuations (RMSF) and the Gyration Radius have been computed using Gromacs. The RMSF has been computed taking into account the last frames of the trajectory where the protein structure is stable (i.e., when the corresponding RMSD reaches a plateau), neglecting the first steps where the protein rearranges its structure in the solvent. The Normal Modes (NMs) and the Principal Component Analysis (PCA) have been performed using the ProDy package (Bakan et al., 2011; Zhang et al., 2021) and these analyses have been carried out with the same frames used to compute the RMSF where the protein has a stable structure. Employing frames where the protein is not stable (i.e., the initial ones) result in a bad description of the normal modes due to large movement of the protein. The PCA has been performed on the first 2,180 amino acids, without considering the flexible region of D3 before the transmembrane portion, which is the most flexible region and could lead into artifacts due to its large motions. The first 20 NMs have been computed and the first 2 associated with low-frequency movements have been described to sample all major motions of the NG2/CSPG4 extracellular portion (Bauer et al., 2019). The Protein Block (PB) analysis has been performed using the PBxplore tool, which computes the possible structural protein prototype during the simulation time (de Brevern et al., 2000). This tool cluster different the 3-dimensional local structure of the protein backbone into Protein Blocks that represent all possible backbone conformations, from alpha-helix to beta-strand and to loops, labeling them with a letter. The probability to find that PB for each residue is drawn in a sequence logo plot and can be quantified by the Number of equivalent PBs, N_eq_, defined as

$$N_{qe}=\exp\left(-\sum_{x=1}^{16}f_{x}\ln\left(f_{x}\right)\right)$$

where f_x_ is the probability of the PB x, given 16 different possible PBs. Higher the N_eq_, higher the conformational flexibility of the residue. The number of hydrogen bonds have been computed using the VMD plugin with donor-acceptor distance of 3.0 Å and an angle cutoff of 20°. Salt-bridges are computed using the VMD plugin with the oxygen-nitrogen cutoff of 3.2 Å and we selected as salt-bridges only those whose distance remains stable under 4.0 Å.

### 2.3 NG2/CSPG4-FGF-FGFR simulation box

Structural details of the interactions between NG2/CSPG4 ectodomain, Fibroblast Growth Factor 2 (FGF-2) and its receptor FGFR-1 were determined by employing the following experimental strategy: (I) for defining the molecular traits of the FGF-FGFR interaction the amino acids in contact between the two proteins were retrieved from the experimental work of Schlessinger et al. (2000); PDB ID 1FQ9); (II) for establishing the structural traits of the FGFR-FGFR dimer we considered the previously described contact sites between FGFRs involved in dimer formation (Schlessinger et al., 2000). The full-length structure of FGFR-1 is not available in literature and only the amino acids from 141 to 365 are available (Schlessinger et al., 2000). The structure of the sequence spanning residues 1–140 was obtained using the AlphaFold3 server (Abramson et al., 2024). The overall structure contains the first 365 amino acids that are predicted to be in the outer region of the cell and amino acids from 374 to 399 intercalated into the cell membrane whereas residues 400–822 belong to the cytoplasmatic domain of the receptor and have not been considering for the present modelling. (III) the full-length structure of FGF2 was not available in literature and only the central region going from amino acid 157–288 has been previously defined (Schlessinger et al., 2000). The structure of the remaining 156 residues belonging the N-terminal have been retrieved using the AlphaFold3 server. This region is modeled as a disordered region. The dimer structure has been obtained by blind docking of two FGF units without taking into account the first 156 disorder amino acids using the SDA webserver (Yu et al., 2015), taking the structure with the highest score for analysis of the FGF (dimer)-NG2/CSPG4 complex, the interaction sites between the NG2/CSPG4 ectodomain and the FGF dimer were been obtained by blind docking using the SDA webserver (Yu et al., 2015) and selecting the highest score structure. The FGF dimer was located into the binding pocket predicted by SDA; the FGFR dimer was inserted into the membrane at a distance of approximately 15 nm between the contact sites of FGF and FGFR, which was determined to lie into the possible range of NG2/CSPG4; according to this model, the FGF-dimer and the FGFR-dimer are not in contact with each other; the topographic scheme obtained for NG2/CSPG4, FGF-dimer, FGFR-dimer and the cell membrane coarse-grained using the Martini Force Field. Water was added to the simulation box and minimization and equilibration simulations were conducted as for the NG2/CSPG4 ectodomain simulation. To reduce the computational time period for simulation of the interaction between the FGF-2-dimer and FGFR-1-dimer, we applied a pulling force to bring closer these two complexes of 100 $\left(kJ/mol\right)/nm$ and a pulling rate of 0.01 nm/ns in the Gromacs package. This force, despite high, is a good compromise between computational effort and structure stability of the proteins, that maintain their secondary and tertiary conformation during the simulation. Under these conditions, simulation was conducted until the FGF-dimer and the FGFR-dimer were in contact each other. A short run of 50ns at 300K has been performed to relax the system without the presence of the external force.

## 3 Results

### 3.1 Intrinsic structural traits of the NG2/CSPG4 ectodomain

**FIGURE 2:**
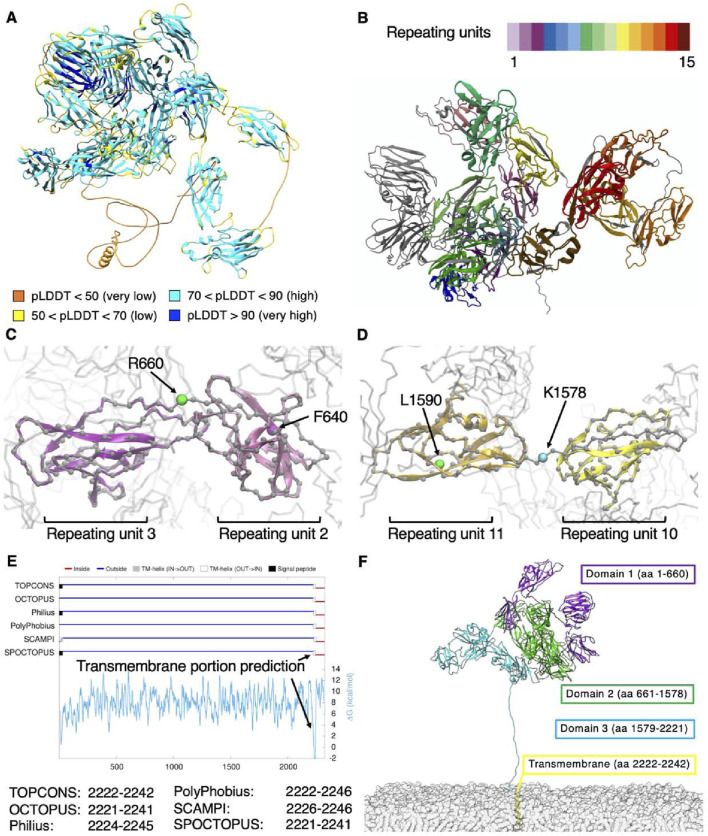
**(A)** NG2/CSPG4 structural model obtained through AlphaFold3 and colored according to the accuracy of prediction using the pLDDT score; **(B)** subdivision in repeats with each repeat having a different color coding; **(C–D)** previous and revised definitions of the subdomains composing the NNG2/CSPG4 ectodomain; **(E)** prediction of the transmembrane portion of NG2/CSPG4 and **(F)** structural overview of the membrane-bound proteoglycan.

The structural traits of NG2/CSPG4 ectodomain have not been previously examined at the atomistic level mainly due to size constraints, while being of paramount importance to better understand its patterns and modes of interaction with extracellular molecules. AlphaFold3 (Abramson et al., 2024) was exploited here to reconstruct the 3D structure of NG2/CSPG4 at atomistic resolution. The derived molecular model with the highest score reports an average pLDDT score of 78.4, where the central portion of the extracellular region of the proteoglycan encompassing the cadherin-like repeating units is modelled with a high accuracy (pLDDT score >80; Figure 2). By contrast, residues at the *N-*terminal end and in the *C-*terminal segment spanning from residue 2,175 to residue 2,322 are predicted with somewhat lower precision. Loops connecting these repeating units and the terminal region of domain D3 are predictable with lower accuracy due to their higher grade of flexibility. In particular, the amino acid sequence corresponding to residues 2,175–2,322 appears poorly folded and flexible. These high-mobile regions contribute to a lowering of the total PTM score of the obtained model, but the models of the single repeats are predicted with better accuracy.

To verify the reliability of the best model obtained by AlphaFold3, we turned to the MobiDB database (Piovesan et al., 2023), which defines for each protein of the human proteome its intrinsically disordered sequences based on the primary structure. Indeed, we could confirm that the disordered residues of NG2/CSPG4 are those spanning from position 1 to 11, i.e., corresponding to the N-terminus, and from position 2,181 to 2,206, corresponding to the end of domain D3 that is contiguous with the transmembrane portion (UniProt ID: Q6UVK1).

Expectedly, residues ranging from positions 2,222 to 2,246 were envisioned to assume an α-helix arrangement characteristic of transmembrane passes of proteins. To confirm this specific feature, we interrogated the IntFold webserver (McGuffin et al., 2023), which gives quality estimates, folds and disorder prediction on the submitted model. The model that was obtained is very similar to the one obtained with AlphaFold3, presenting an overall pLDDT score of 74.8 and pTM score of 0.527. The IntFold-derived model shows an overall globular shape of NG2/CSPG4 with a radius of gyration of 5.076 nm, which would be compatible with that derived from the AlphaFold3 prediction (i.e., a value of 5.13). AlphaFold3 analyses also largely confirm the structural features reported experimentally in the PDB database [PDB IDs: 7ML7, 7N8X and 7N9Y (Jiang et al., 2022)] for stretches L411-N550 and L411-P548. The comparison of these structures with the AlphaFold model demonstrates a strong agreement between the secondary and tertiary structures, as reported in Supplementary Figure. S1A.

Considering all the above comparison, we can assess that the protein model contains high confidence regions, i.e., the repeating units, that are similar each other in their secondary and tertiary structures and that are in good agreement with already known experimental structures. Low confidence regions belong to coils connecting the repeating units and that are responsible of the lower overall score of the model. Moreover, the sequence of coil in D3 contains numerous disorder-promoting amino-acids (AARTEAGKPESSTPTGEPGPMASSPEPAVAKGGFLSFLEANM), confirming the lower score of AlphaFold. The PAE analysis shows that repeating units are well predicted, while it seems that distances between them are not well predicted. This is due to the presence between each repeating unit of a short-disordered coil that gives to the entire protein its flexibility. In order to test the model under physiological conditions, we performed Molecular Dynamics (see next paragraph) and computed the distances between residues. We obtained that the structure from simulations look very similar to the PAE distribution where repeating units are well defined and the flexibility between them is preserved, see Supplementary Figure. S1B, C.

By performing a deep visual inspection at the atomistic level of each repeating unit to ensure consistency with the protein’s secondary and tertiary structures, we observed that the last amino acid of domain D1, i.e., residue F640, falls within repeat 2, while the last amino acid of domain D3, i.e., residue L1590, resides within repeat 11 (Figures 2C, D). In order to gain a finer partitioning of each of the domains delineated by Staub et al. (2002), we modified the classical definition of the NG2/CSPG4 cadherin-like domains, such as to embody in each domain an entire repeat. According to this alternative subdomain partition, the three key domains would stretch from residues 1–660 for D1, from 661–1,578 for D2 and from 1,579–2,221 for D3. Moreover, when we consulted the TOPCONS webserver (Tsirigos et al., 2015) to predict the transmembrane domain of NG2/CSPG4, we found that different algorithms identified similar sequences suggesting that residues 2,222 to 2,242 invariably belong to the transmembrane portion (Figure 2E).

We next defined the transmembrane sequence as including residues at positions 2,222 to 2,242, in accordance with the prediction available through TOPCONS. Remaining residues were predicted to be largely unfolded and to be structuring the cytoplasmatic domain. The new definition of the NG2/CSPG4 domains and the corresponding revised positions of the CSPG4 repeats are reported in Table 1.

**TABLE 1:** Subdivision of domains and CSPG repeats of NG2 accordingly to this work.

	Domain 1			Domain 2								Domain 3					
Repeat		1	2	3	4	5	6	7	8	9	10	11	12	13	14	15	
Amino-acid	1–427	428–523	553–645	662–764	783–878	898–989	1,018–1,110	1,126–1,216	1,238–1,337	1,356–1,449	1,473–1,563	1,581–1,679	1704–1803	1832–1924	1941–2029	2038–2,147	2,148–2,221

The final overall structure of the proteoglycan including the membrane anchoring arrangement is reported in Figure 2F. The terminal portion of the D3 domain was stretched out to perform simulations with the core of the proteoglycan far from the cell membrane, such as to avoid a possible bias introduced by a membrane contact at the initial steps of the simulation. The manual re-arrangement of the C-terminal is required because the model predicted both by AlphaFold3 (Abramson et al., 2024) and IntFold webserver (McGuffin et al., 2023) pack the transmembrane region close to the protein core, without taking into account the excluded volume from the cellular membrane. Full-length reconstruction of the proteoglycan reveals that is mainly made up by β-sheets connected by loops and coils, while few regions of the molecule contain α-helices. The three domains appear closely packed, with domain D1 pointing outward and domain D3 angled towards the cell membrane, thereby obtaining an overall globular shape of the proteoglycan with a tail protruding out at the end of domain D3.

It is well known that NG2/CSPG4 is a highly glycosylated protein and that its fully glycosylated form can reach an apparent molecular weight >500 kDa (Girolamo et al., 2013), while the molecular weight of NG2/CSPG4 is 250 kDa. This strong increase in the molecular weight of the fully glycosylated form is due to the high number of N- and O-glycosylation sites that span the overall length of the protein, as predicted by NetNGlyc-1.0 and NetOGlyc-4.0 webservers (Steentoft et al., 2013). The complete list of N- and O-glycosylation sites is reported in Supplementary Table. S1 and in Supplementary Figure. S1D.

### 3.2 Predicted structural dynamics of the NG2/CSPG4 extracellular portion

The molecular dynamics of the NG2/CSPG4 ectodomain were analyzed by adopting both full-atomistic and Coarse-Grained molecular dynamics simulations. Due to the high number of atoms composing this portion of NG2/CSPG4, full-atomistic simulations are computationally overloaded and cannot reach long timescales. Coarse-Grained models offer an elegant method to decrease the number of atoms to be simulated, thereby reducing the computational efforts and allowing to simulate systems composed by a larger number of atoms for longer timescales. Here, we employed the Martini2 force field (Chakraborty et al., 2021) to simulate the dynamic properties of the NG2/CSPG4 ectodomain, since this model has shown satisfactory performances when applied to glycoprotein dynamics (Chakraborty et al., 2021) and to the interaction of proteins with the cell membrane (Mahmood et al., 2021). To validate the CG model with respect to the full-atomistic one, we compared the obtained simulation results considering the extracellular portion of the proteoglycan in the absence of the cell membrane. In both models, i.e., with and without cell membrane, only small changes with respect to the initial structure were observed and these were mainly confined to the highly flexible *N-* and *C-*terminal ends of the proteoglycan’s extracellular portion.

**FIGURE 3:**
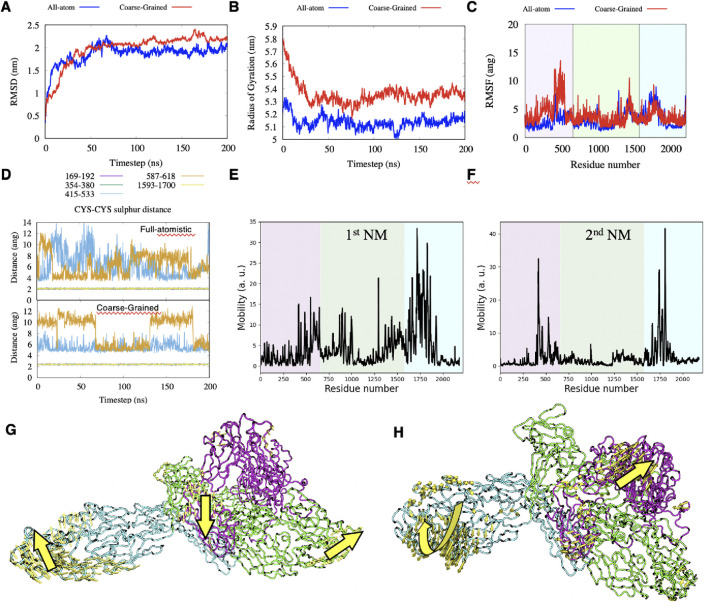
**(A–C)** comparison between full-atomistic and Coarse-Grained simulations of the RMSD, Radius of Gyrations and RMSF, respectively. **(D)** distance between sulfur atoms during the full-atomistic and Coarse-Grained simulation time when considered for all pairs of cysteine-cysteine appositions. **(E–H)** mobility plots of the first two Normal Modes and graphical representations of these modes.

The RMSD and Gyration radius suggested that the protein reaches a stable configuration after 100 ns and that it is rather compact. The CG model gave a slightly higher value for the gyration radius (Figures 3A, B). According to the RMSF plot, the ectodomain of NG2/CSPG4 encompasses three main flexible regions (Figure 3C): (I) the stretch of residues 413–431, which corresponds to the *N-*terminal portion of D1. This region is particularly important for biological functions because it contains signaling sites and binding sites for growth factors, *Clostridioides difficile* secretes exotoxins (TcbdA and TcdB (Chen et al., 2021; Jiang et al., 2022)). (II) the stretch of residues 1,292–1,300 and 1,367–1,469 of repeats 8 and 9 residing within D2; and (III) the stretch of residues1660-1866, which comprises residues from the end of repeat 11 and residues extending to repeat 13 of the D3 domain.

The number of hydrogen bonds in both replicas remains constant to an average value of 440 after the first 100ns of simulation, see Supplementary Figure. S1E, suggesting that results are reproducible. Among all possible 221 amino acids pairs that can in principle form a salt-bridge, we observed that only 4 pairs have the oxygen-nitrogen distance permanently under 4.0Å during the simulation time, see Supplementary Figure. S1F: GLU195-ARG167; ASP1157-ARG1,035; GLU1486-LYS1,578 and ASP1860-ARG1826.

The cadherin-like repeats of the NG2/CSPG4 ectodomain are reciprocally stabilized through three disulfide bonds at C169-C192 and C345-C380 within domain D1 and C1593-C1700 within domain D3. These bonds are found to be stable both using full-atomistic and CG simulations (Figures 3D, E). Other two pairs of cysteine residues are found in close contacts within domain D2 (C415-C533 and C587-C618), but they do not seem to consistently form disulfide bonds in both models. The first five Normal Modes (NMs) have been computed using the ProDy package (Zhang et al., 2021) and they represent low-frequency collective movements of the protein and could be related to functional properties of the proteoglycan. We observed that the first two NMs are large enough to describe the main motions of NG2/CSPG4, while higher order NMs show smaller movements that can be tracked back to the first two NMs (Figures 3E, F; see also Methods). The main and most pronounced movement from the first NM is given by the stretching of the segments involving the sequences corresponding to residues 792–801, 865–872, 889–903 and 925–930, of domain D2, and residues 1,622–1,644, 1,659–1837 of domain D3. At the same time, the amino acid sequence spanning the residues 579–622 within domain D1 intersects like an asymmetric-stretching mode (Figure 3G). The second motion mode is of less intensity with respect to the first one and deals with simultaneous twisting of the outer region of domain D3, accompanied by the stretch of the segment localized between domains D1 and D3 and observed for the first mode (Figure 3H).

The secondary structure of the extracellular portion of NG2/CSPG4 computed using the DSSP package (Kabsch and Sander, 1983; Touw et al., 2015) is maintained through all simulations, confirming that the cadherin-like repeats show a high degree of stability, while loops and coils connecting the repeats remains disordered. This analysis corroborates that the NG2/CSPG4 ectodomain is mainly made up by β-sheets connected by coils and loops with short α-helices and the N- and C-terminal portions remaining disordered (Supplementary Figure. S2).

### 3.3 Interactions of the NG2/CSPG4 ectodomain with the outer cell membrane surface and with extracellular ligands

**FIGURE 4:**
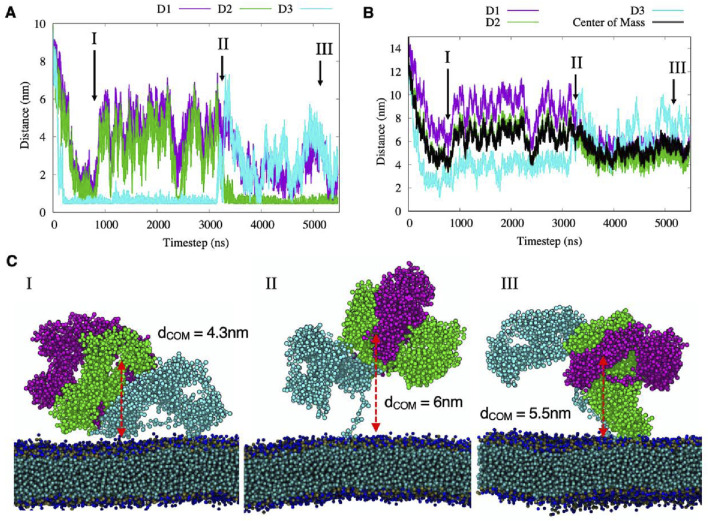
**(A–B)** the minimum and the center of mass distances between each domain of NG2/CSPG4 and the membrane surface, where black line represents the Center of Mass of the three domains. Different protein-membrane configurations are labeled with roman numbers and their graphical representations are reported in panel **(C)**.

The 500ns full-atomistic simulation was performed also with the transmembrane portion of NG2/CSPG4 inserted into the cell membrane for a total of 3′754′099 atoms. The hydrophobic residues close to the transmembrane domain make persistent contacts with the membrane surface, bringing the D2 and D3 domains closer to it, while domain D1 remains consistently pointed outward with respect to the cell membrane. Secondary structure of the proteoglycan seems to remain stable during all simulation time and it is comparable to the simulations performed without the cell membrane. To then evaluate the putative time-resolved configurational rearrangements of the NG2/CSPG4 ectodomain when protracted during longer simulation periods, we relied upon the CG model and performed two independent 5.5 µs long simulations. Figures 4A, B reports the minimum distance between each atom of each of the domains touching the cell membrane, and the average distance of the center of each domain with respect to the cell membrane surface for simulations performed at long timescales.

We observed that in the first hundreds of nanoseconds both domain D2 and domain D3 contacted the cell membrane without making any persistent, more stable attachment and did so remaining at a distance from the center of mass of 4 nm (Figure 4C; Configuration I). This molecular motion appeared to be generated by the interaction of hydrophobic residues of the flexible segments of domain D3 with the cell membrane surface. Then, the protein displaced away from the membrane surface to reach an approximate distance of 6 nm from the surface, while maintaining domain D3 in contact with the membrane. In this structural arrangement, reported in Configuration I of Figure 4, domain D1 appeared completely exposed to the solvent and the D2 domain partially exposed to regain contact with the membrane after 2.3µs. This new contact could be simulated for a short period of time, suggesting that it may be a sporadic interaction. At 3.2µs, the D3 domain desorbs from the membrane (Figure 4C; Configuration II) and the NG2/CSPG4 ectodomain remains free to float over the membrane with the unfolded *C-*terminal region stretched out.

When assuming this molecular arrangement, the protein core is predicted to rotate around its principal axis while the D2 domain touches the membrane (Figure 4C; Configuration III). In this specific molecular arrangement, NG2/CSPG4 has an average distance of its center of mass of 5.5 nm, with the D2 domain partially exposed to the solvent while domain D3 is totally exposed. A similar behavior is observed in the second replica of the CG simulation where both the D2 and D3 domains sporadically interact with the membrane without making persistent contacts with it and leaving the D1 domain exposed to possible binding with other proteins or molecules (Supplementary Figure S3). These findings suggest that the D1 domain could be the preferred interaction region for other proteins and molecules, while the D2 and D3 domains are partially hidden, as suggested by Tan et al (2005). The calculation of the Solvent Accessible Surface Area (SASA) computed for the full atomistic simulation confirms that the average area per residue in domain D1 is equal to 0.5664 nm^2^, for domain D2 is 0.5251 nm^2^ and for domain D3 is 0.5279 nm^2^, suggesting that D1 is the more probable NG2/CSPG4 binding site for extracellular molecules. This conclusion is supported by previous experimental data on the binding of antigens against the three domains. Ughrin et al. (2003) showed that when NG2/CSPG4 is an integral membrane protein, the D1 is the most available for interactions while D3 is the least available due to the presence of the membrane.

The effect of intercalation of CSPG4 into the membrane does not affect the overall mobility of the protein extracellular region, as reported by the RMSF measurement (Supplementary Figure S4A). However, we observed contact between repeating units 1 and 10 that is not present in the free-protein case, but that has negligible effects on the overall conformational arrangements of the protein and its dynamics (Supplementary Figure S4B). Moreover, the “Normal Modes” analysis shows a similar dynamic to the atomistic case, where major motions are localized in the extremities of domains D1 (*N-*terminal) and D3 (*C-*terminal; Supplementary Figure S4C, D). The protein flexibility was also determined by the “Protein Block” analysis, which describes the local variability of all amino acids secondary structure during the simulation time (Supplementary Figure S4E). The Number of equivalent structures (Neq) computed on the full atomistic trajectory shows that the *N-*terminal segment and the more C-terminal stretch of the D3 domain (aa 2,180–2,221) are the most flexible regions of this domain, while sequences inside the repeating units appear more rigid (Supplementary Figure S4F). These findings suggest that the tertiary structure of NG2/CSPG4 remains mostly compact with the three domains interacting with each other and leaving the D1 domain more exposed to the solvent. The structural arrangement causes the D3 domain to be less accessible for molecular interactions.

The accessibility of different domains by other biological entities is of paramount importance to design molecules, proteins and antibodies that target NG2/CSPG4. Here, we observed that D1 is always accessible from the extracellular side, while D2 and D3 are partially hidden due to the flexible region at the C-terminal that moves the protein core close to the membrane. However, the interaction with the membrane can change during time exposing different sides of the protein, as shown in Figure 4.

Interestingly, the flexible region at the end of D3 connecting the core (aa 1–2,179) with the cellular membrane (aa 2,180–2,221) could be an important element to define interactions with neighboring proteins. In the effort to quantify the closeness of proteins to be able to interact with NG2/CSPG4, we computed the potential area that NG2/CSPG4 can span when intercalated into the cellular membrane. Assuming the protein core as a rigid body, this flexible region could span a region of approximately 440 nm^2^, considering the maximum radius of 21 nm from the membrane to the farthest region of NG2/CSPG4 (Supplementary Figure S5A). This suggest that another membrane protein needs to be in that range to interact with NG2/CSPG4.

It is recognized that the Martini model (both Martini 2 and Martini3) tend to overestimates the protein-protein interactions and some solutions are currently under development (Soni et al., 2024; Thomasen et al., 2024). In this case, this leads to excessive adhesion of flexible loops connecting domains, in particular D2 and D3, obtaining a less extended tertiary structure with domains more close each other. This is supported also by the calculation of the RMSD of the residues in the range 1–2,180 and results are reported in Supplementary Figure S5B. We can observe that by neglecting the long coil in D3, the RMSD of the Martini model reaches a plateau in few ns suggesting that the structure is very stable due to the protein-protein strong interactions, while in the atomistic case the RMSD curves are very similar each other. However, the comparison between atomistic and Coarse-Grained structures, reported in Supplementary Figure S3C, gives a good agreement, suggesting that this overestimation has a minor influence on the overall results and that the Martini 2 model remains a reliable choice for large membrane-containing systems, as demonstrated in prior studies.

### 3.4 Predicted docking mechanics of the NG2/CSPG4 ectodomain in FGF-2-FGFR-1 binding

Among the numerous molecular interactions that NG2/CSPG4 engages with extracellular ligands, the glycosaminoglycan-independent binding to growth factors and their receptors is the most convincingly described one (Nishiyama et al., 1996; Goretzki et al., 1999; Grako et al., 1999; Stallcup, 2002; Cattaruzza et al., 2013; Zhen et al., 2018). There is solid experimental evidence demonstrating an interaction of NG2/CSPG4 with FGFs and their receptors and these interactions have previously been hypothesized to promote the formation of trimeric NG2/CSPG4-FGF-FGFR complexes more optimally triggering intracellular signaling (Goretzki et al., 1999; Cattaruzza et al., 2013; Zhen et al., 2018).

However, how exactly the NG2/CSPG4 ectodomain would physically bring about these putative trivalent interactions and contribute to the combined dimerization of ligand and receptor have remained unknown. The possibilities that have been hypothesized thus far (Goretzki et al., 1999) are: 1) that NG2/CSPG4 promotes FGF dimerization by linking to one of the FGF monomers or both simultaneously; 2) that sequestering of FGFs by NG2/CSPG4 facilitate the interaction of the growth factors with the cognate receptors; and 3) that the NG2/CSPG4 interaction with FGF receptors induces conformational changes of the receptors that favor ligand binding and their constitutive dimerization (consistently with what has been proposed to be NG2/CSPG4’s ability to modulate “receptor activation”).

To address these different possibilities by leveraging on computational modeling predictions, we have examined the interactions between NG2/CSPG4, FGF-2 and its receptor FGFR-1. In this kind of simulation, the aim was only to test if the NG2/CSPG4 could be the co-receptor of for the FGF-2 and if it is able to transport it to the receptor without interacting with it. The evaluation of the obtained structure of the complex NG2/CSPG4-FGF-FGFR in a more accurate manner will require an enhanced sampling technique of all possible structures, but this was not the main goal of these simulations and it will be a topic for future studies. The construction of the simulation box containing one NG2/CSPG4, two FGF-2 molecules associating to form a dimer, two FGFR-1 molecules anchored to the membrane in association to also form a dimer and the cell membrane is reported in Methods. FGFR-1 was docked at approximately 15 nm from the FGF-2-dimer, considering the maximum radius that CSPG4 can span over the cellular membrane as previously defined (Supplementary Figure 4). Our docking analysis shows that the FGF-2-dimer is stabilized by the disulfide bond C95-C95 (C229-C229 accordingly to the numbering scheme employed here where the first 134 residues are taken into account) where cysteines have a distance of 0.9 nm, while the C77-C77 (C211-C211 in this work) disulfide bond is not observed due to their distance of 3.4 nm (Supplementary Figure 6). This is in good agreement with the recent work of Lolicato et al. (2024) reporting the formation of the C95-C95 disulfide bond in the FGF-2-dimer spanning 0.9 nm, whereas the C77-C77 disulfide bond stretch was determined to be about 3.5 nm.

**FIGURE 5:**
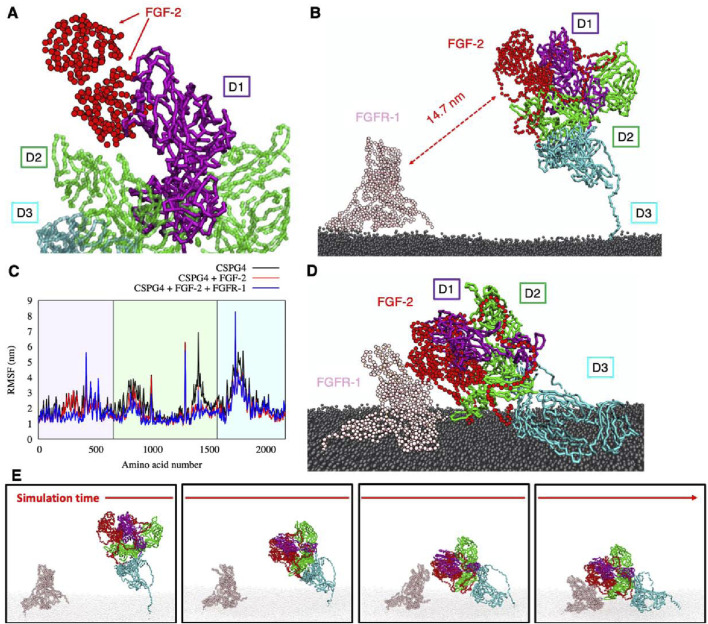
**(A)** binding of FGF-dimer and NG2/CSPG4 as obtained from SDA package; **(B)** the simulation box containing NG2/CSPG4, FGF-dimer, FGFR-dimer and the cellular membrane (water not shown for clarity); **(C)** RMSF comparison of CSPG4 before the binding with FGF-dimer (black curve), after the binding with FGF-dimer (red curve) and after the binding of FGF-dimer with FGFR-dimer (blue curve); **(D)** final configuration of the system; **(E)** four snapshots of the simulation showing the binding of FGF-dimer with FGFR-dimer and the bending of the D3 flexible region. Each protein has a different color, reported in panels **(B, D)**.

In the first part of the simulation, NG2/CSPG4 and the FGF-2-dimer are observed to come in direct contact, whereas the FGFR-1-dimer does not appear to interact (Figures 5A, B). The FGF-2-dimer is observed to interact with the D1 domain, as predicted by SDA docking, and during the simulation to also bind to the D2 domain, due to its proximity. The overall intrinsic mobility of NG2/CSPG4 remains unaltered, except for the residues constituting the FGF-2-dimer binding sites within the D1 domain, as reported in Table 2 **,** and the residues of domain D2, where the interaction with FGF-2 seems to exert a stabilizing effect (i.e., lower RMSF values). By contrast, the intrinsic mobility of the D3 domain does not seem to be affected (Figure 5C).

**TABLE 2:** Contact sites between the different units of each NG2/CSPG4/FGF-2/FGFR-1 complex.

Complexes 1–2	Amino acids in complex 1	Amino acids in complex 2	Source
FGFR - FGFR	A167, V168, P169, A170, A171, K172, T173, D218, S219	A167, V168, P169, A170, A171, K172, T173, D218, S219	Ibrahimi et al. (2005)
FGF - FGFR	F159, K160, G170, G171, Q198, A199, D200, D201, N243, N244, Y245	E162, K163, H166, A167, V168, P169, R250, P252, P283, Q284, P285, H286, A314, G315, V316, T319, D320, G344, N345, S346, I347, G348	Ibrahimi et al. (2005)
FGF – FGF	G149, E201, R202, C229, V230, T231	G149, E201, R202, C229, V230, T231	SDA Yu et al. (2015) Lolicato et al. (2024)
FGF - CSPG4	V258, T272, G273, P274, G275, Q276, A278, I279	L481, E482, I485, P486, G487, A488, Q489, A490	SDA Yu et al. (2015)

The contact between the FGF-2-dimer and the FGFR-1-dimer takes place after the application of an external force that drives the interaction, reducing the computational cost and using the distance between the known interacting residues (Ibrahimi et al., 2005) (Table 2) as pulling groups in GROMACS. The extracellular portion of NG2/CSPG4 gets closer to FGFR-1 due to the high flexibility of the C-terminal region (aa 2,180–2,221) in domain D3, which is of fundamental importance in this kind of interaction. FGFRs show a minor intrinsic mobility with respect to their starting positions, and they gradually approach the NG2/CSPG4-FGF-2 complex. After a stable contact between FGF-2 and FGFR-1 has been established, the oligomeric complex stabilizes through small sidechains movements that do not affect the binding site and the stability of the complex. NG2/CSPG4 does not seem to interact directly with the FGFR-1-dimer, suggesting that the prevalent interaction of NG2/CSPG4 with the FGF-dimer traps the FGFR-1 dimer, facilitating its binding to the ligand dimer. Such interpretation of the observed computational model would then suggest that the role played by NG2/CSPG4 in this context is to bring in juxtaposition ligand molecules and the receptors, as previously hypothesized (Goretzki et al., 1999; Cattaruzza et al., 2013; Zhen et al., 2018).

## 4 Discussion

Through different *in silico* computational methods we have pioneered the atomistic simulation of the topographical and conformational dynamics of the extracellular portion of the transmembrane proteoglycan NG2/CSPG4, focusing as a proof-of-concept on its putative function as growth factor co-receptor. Thus far, *in silico* investigations of the proteoglycans have been strongly hampered by its excessive size and structural complexity and therefore analyses as those previously performed on the proteoglycan CD44 (Nguyen et al., 2016; Lintuluoto et al., 2021) have been challenging. By leveraging on multi-scale computational procedures based on both full-atomistic and coarse-grained molecular dynamics, we simulated NG2/CSPG4 for extended timescales under physiological conditions. The atomistic analyses of the repeating units composing the overall protein structure allowed us to reshape the three-domain arrangement of the proteoglycan’s extracellular segment previously established based on the primary amino acid sequence (Nishiyama et al., 1996; Pluschke et al., 1996; Tamburini et al., 2019), such as to more accurately delineate the extension of structural-functional segments of the proteoglycan.

By simulating the intercalation of the transmembrane region into a lipid bilayer of a cell membrane, our predictions show that the D1 domain is always accessible from the extracellular side, while D2 and D3 are partially hidden due to the flexible region at the *C-*terminal that moves the protein core close to the membrane. This effect of excluded volume due to the interaction with the membrane will influence the accessibility of sites of NG2/CSPG4 ectodomain or the tendency of the linked proteins to oligomerize (Rivas and Minton, 2024). This finding may implicate a spatial functional partition of the D1-D3 domains of the proteoglycan (Figure 1). According to such model, the D1 domain seems primarily responsible for the sequestering of soluble ligands, the central D2 one preferentially engaged in the anchoring to the ECM through linkage to collagen type VI microfilaments (Stallcup et al., 1990; Nishiyama and Stallcup, 1993; Burg et al., 1997; Tillet et al., 2002; Cattaruzza et al., 2013; Sardone et al., 2016) and basement membrane constituents, such as perlecan (Tang et al., 2018), and the cell membrane-proximal D3 domain mediating the association of NG2/CSPG4 with components known to promote cell-cell interactions, such as galectin-3 and α3β1 α4β1 integrins (Iida et al., 1995; Tillet et al., 2002; Fukushi et al., 2004; Wen et al., 2006; Stallcup, 2017; Iida et al., 1995; Tillet et al., 2002; Fukushi et al., 2004; Wen et al., 2006; Stallcup, 2017). Moreover, the flexible *C-*terminal region seems to account for the predicted bending of the NG2/CSPG4 ectodomain toward the cell membrane and may thereby facilitate the interaction with proteins spanning a wide region of the membrane surface, including both intercalated and membrane-associated ones. To what extent this flexibility is autonomously governed by the extracellular portion of the proteoglycan, or maybe influenced by the dynamics of cytoplasmic upon its interaction with the cytoskeleton, remains to be determined.

To challenge the model predicting maximal availability of the D1 domain for binding to extracellular soluble ligands, we simulated the interaction of FGF-2 with NG2/CSPG4 in its monomeric and dimerized form and further contemplated the previously proposed complex formation of NG2/CSPG4 with the FGFR-1 homodimer. When the putative FGF-2 docking receptor function of the proteoglycan was simulated, it was observed that NG2/CSPG4 could bind single and dimeric forms of the growth factor but did not seem to interact with single or dimeric arrangements of FGFR-1. Conversely, the bending of the NG2/CSPG4 ectodomain toward the cell membrane, as observed, is predicted to facilitate the release of sequestered growth factor ligands towards their receptors, thereby supporting the proteoglycan’s role as a growth factor co-receptor from a mechanistic perspective. It is worth noting that our simulations suggest that the NG2/CSPG4 ectodomain bends autonomously toward the cell membrane without the application of an external force, as indicated by the apparent ability of the protein to computationally move into the solvent with a wide interacting range. Our analyses further suggest that FGF-2-interactive region of NG2/CSPG4 is located inside a D1/D2 pocket, leaving a large portion of D1 domain still available for the binding with other extracellular ligand, such as other growth factors (i.e., PDGF-AA and IGFs) or other signaling molecules, such as angiostatin (Goretzki et al., 2000), and signaling receptors, such as FLT3 (Lopez-Millan et al., 2024). Regarding this latter interaction, the findings originated through this computational analysis define the putative structural basis for the dynamics of the FLT3-NG2/CSPG4 binding. Through the flexibility of NG2/CSPG4 to bend towards the cell membrane, the upper D1 domain of proteoglycan is physically brought into contact with the FLT3 receptor, which is a significantly shorter molecule (Figure 1). The fact that molecular interactions involving the D2 and D3 domains have only been demonstrated using isolated recombinant fragments encompassing such domain leaves open whether these interactions are taking place with the intact NG2/CSPG4 ectodomain. If ensuing, it would be particularly valuable to understand whether molecular interactions with these domains depend on the flexible nature of the proteoglycan and whether they may be dictated by specific configurations assumed by those specific regions or the protein.

Overall, the computational approach proposed here demonstrates the possibility to study the secondary and tertiary structures of large membrane-intercalated proteins, approach their dynamic interactions with cell surface components and predict their putative function as co-receptors for signaling molecules. Computational analyses for extended timescales combined with *in silico* prediction methods are therefore believed to be the key to gain significant structural-functional information about the molecular interactions engaged by NG2/CSPG4 and similar proteoglycans to back-up mechanistically their biological and pathological roles.

## Data Availability

The raw data supporting the conclusions of this article will be made available by the authors, without undue reservation.

## References

[B1] Abraham M. J. Murtola T. Schulz R. Páll S. Smith J. C. Hess B. (2015). GROMACS: high performance molecular simulations through multi-level parallelism from laptops to supercomputers. SoftwareX 1–2, 19–25. 10.1016/j.softx.2015.06.001

[B2] Abramson J. Adler J. Dunger J. Evans R. Green T. Pritzel A. (2024). Accurate structure prediction of biomolecular interactions with AlphaFold 3. Nature 630, 493–500. 10.1038/s41586-024-07487-w 38718835 PMC11168924

[B3] Bakan A. Meireles L. M. Bahar I. (2011). ProDy: protein dynamics inferred from theory and experiments. Bioinformatics 27, 1575–1577. 10.1093/bioinformatics/btr168 21471012 PMC3102222

[B4] Bauer J. A. Pavlović J. Bauerová-Hlinková V. (2019). Normal mode analysis as a routine part of a structural investigation. Mol. Basel Switz. 24, 3293. 10.3390/molecules24183293 PMC6767145 31510014

[B5] Burg M. A. Nishiyama A. Stallcup W. B. (1997). A central segment of the NG2 proteoglycan is critical for the ability of glioma cells to bind and migrate toward type VI collagen. Exp. Cell Res. 235, 254–264. 10.1006/excr.1997.3674 9281375

[B6] Cattaruzza S. Ozerdem U. Denzel M. Ranscht B. Bulian P. Cavallaro U. (2013). Multivalent proteoglycan modulation of FGF mitogenic responses in perivascular cells. Angiogenesis 16, 309–327. 10.1007/s10456-012-9316-7 23124902 PMC3656602

[B7] Cengiz C. Bulut S. Boyacioglu A. S. Kuzu M. A. (2017). Nerve/Glial antigen 2: a novel target for anti-tumor therapy in colorectal cancer. Digestion 96, 60–66. 10.1159/000478853 28715802

[B8] Chakraborty S. Wagh K. Gnanakaran S. López C. A. (2021). Development of Martini 2.2 parameters for N-glycans: a case study of the HIV-1 Env glycoprotein dynamics. Glycobiology 31, 787–799. 10.1093/glycob/cwab017 33755116 PMC8351497

[B9] Chen P. Zeng J. Liu Z. Thaker H. Wang S. Tian S. (2021). Structural basis for CSPG4 as a receptor for TcdB and a therapeutic target in Clostridioides difficile infection. Nat. Commun. 12, 3748. 10.1038/s41467-021-23878-3 34145250 PMC8213806

[B10] de Brevern A. G. Etchebest C. Hazout S. (2000). Bayesian probabilistic approach for predicting backbone structures in terms of protein blocks. Proteins 41, 271–287. 10.1002/1097-0134(20001115)41:3<271::aid-prot10>3.0.co;2-z 11025540

[B11] de Jong D. H. Singh G. Bennett W. F. D. Arnarez C. Wassenaar T. A. Schäfer L. V. (2013). Improved parameters for the Martini coarse-grained protein force field. J. Chem. Theory Comput. 9, 687–697. 10.1021/ct300646g 26589065

[B12] Fukushi J. Makagiansar I. T. Stallcup W. B. (2004). NG2 proteoglycan promotes endothelial cell motility and angiogenesis via engagement of galectin-3 and alpha3beta1 integrin. Mol. Biol. Cell 15, 3580–3590. 10.1091/mbc.e04-03-0236 15181153 PMC491820

[B13] Garusi E. Rossi S. Perris R. (2012). Antithetic roles of proteoglycans in cancer. Cell. Mol. Life Sci. CMLS 69, 553–579. 10.1007/s00018-011-0816-1 21964924 PMC11114698

[B14] Girolamo F. Dallatomasina A. Rizzi M. Errede M. Wälchli T. Mucignat M. T. (2013). Diversified expression of NG2/CSPG4 isoforms in glioblastoma and human foetal brain identifies pericyte subsets. PLOS ONE 8, e84883. 10.1371/journal.pone.0084883 24386429 PMC3873429

[B15] Goretzki L. Burg M. A. Grako K. A. Stallcup W. B. (1999). High-affinity binding of basic fibroblast growth factor and platelet-derived growth factor-AA to the core protein of the NG2 proteoglycan. J. Biol. Chem. 274, 16831–16837. 10.1074/jbc.274.24.16831 10358027

[B16] Goretzki L. Lombardo C. R. Stallcup W. B. (2000). Binding of the NG2 proteoglycan to kringle domains modulates the functional properties of angiostatin and plasmin(ogen). J. Biol. Chem. 275, 28625–28633. 10.1074/jbc.M002290200 10889192

[B17] Grako K. A. Ochiya T. Barritt D. Nishiyama A. Stallcup W. B. (1999). PDGF (alpha)-receptor is unresponsive to PDGF-AA in aortic smooth muscle cells from the NG2 knockout mouse. J. Cell Sci. 112 (Pt 6), 905–915. 10.1242/jcs.112.6.905 10036240

[B18] Huang J. Rauscher S. Nawrocki G. Ran T. Feig M. de Groot B. L. (2017). CHARMM36m: an improved force field for folded and intrinsically disordered proteins. Nat. Methods 14, 71–73. 10.1038/nmeth.4067 27819658 PMC5199616

[B19] Ibrahimi O. A. Yeh B. K. Eliseenkova A. V. Zhang F. Olsen S. K. Igarashi M. (2005). Analysis of mutations in fibroblast growth factor (FGF) and a pathogenic mutation in FGF receptor (FGFR) provides direct evidence for the symmetric two-end model for FGFR dimerization. Mol. Cell. Biol. 25, 671–684. 10.1128/MCB.25.2.671-684.2005 15632068 PMC543411

[B20] Iida J. Meijne A. M. Spiro R. C. Roos E. Furcht L. T. McCarthy J. B. (1995). Spreading and focal contact formation of human melanoma cells in response to the stimulation of both melanoma-associated proteoglycan (NG2) and alpha 4 beta 1 integrin. Cancer Res. 55, 2177–2185. 7743521

[B21] Ilieva K. M. Cheung A. Mele S. Chiaruttini G. Crescioli S. Griffin M. (2017). Chondroitin sulfate proteoglycan 4 and its potential as an antibody immunotherapy target across different tumor types. Front. Immunol. 8, 1911. 10.3389/fimmu.2017.01911 29375561 PMC5767725

[B22] Jiang M. Shin J. Simeon R. Chang J.-Y. Meng R. Wang Y. (2022). Structural dynamics of receptor recognition and pH-induced dissociation of full-length Clostridioides difficile Toxin B. PLoS Biol. 20, e3001589. 10.1371/journal.pbio.3001589 35324891 PMC8982864

[B23] Jo S. Kim T. Iyer V. G. Im W. (2008). CHARMM-GUI: a web-based graphical user interface for CHARMM. J. Comput. Chem. 29, 1859–1865. 10.1002/jcc.20945 18351591

[B24] Kabsch W. Sander C. (1983). Dictionary of protein secondary structure: pattern recognition of hydrogen-bonded and geometrical features. Biopolymers 22, 2577–2637. 10.1002/bip.360221211 6667333

[B25] Lee J. Patel D. S. Ståhle J. Park S.-J. Kern N. R. Kim S. (2019). CHARMM-GUI membrane builder for complex biological membrane simulations with glycolipids and lipoglycans. J. Chem. Theory Comput. 15, 775–786. 10.1021/acs.jctc.8b01066 30525595

[B26] Lintuluoto M. Horioka Y. Hongo S. Lintuluoto J. M. Fukunishi Y. (2021). Molecular dynamics simulation study on allosteric regulation of CD44-hyaluronan binding as a force sensing mechanism. ACS Omega 6, 8045–8055. 10.1021/acsomega.0c05502 33817464 PMC8014924

[B27] Lolicato F. Steringer J. P. Saleppico R. Beyer D. Fernandez-Sobaberas J. Unger S. (2024). Disulfide bridge-dependent dimerization triggers FGF2 membrane translocation into the extracellular space. eLife 12, RP88579. 10.7554/eLife.88579 38252473 PMC10945597

[B28] Lopez-Millan B. Rubio-Gayarre A. Vinyoles M. Trincado J. L. Fraga M. F. Fernandez-Fuentes N. (2024). NG2 is a target gene of MLL-AF4 and underlies glucocorticoid resistance in MLL-r B-ALL by regulating NR3C1 expression. Blood 144, 2002–2017. 10.1182/blood.2023022050 39093982

[B29] MacKerell A. D. Bashford D. Bellott M. Dunbrack R. L. Evanseck J. D. Field M. J. (1998). All-atom empirical potential for molecular modeling and dynamics studies of proteins. J. Phys. Chem. B 102, 3586–3616. 10.1021/jp973084f 24889800

[B30] Mahmood Md. I. Poma A. B. Okazaki K. (2021). Optimizing gō-MARTINI coarse-grained model for F-bar protein on lipid membrane. Front. Mol. Biosci. 8, 619381. 10.3389/fmolb.2021.619381 33693028 PMC7937874

[B31] McGuffin L. J. Edmunds N. S. Genc A. G. Alharbi S. M. A. Salehe B. R. Adiyaman R. (2023). Prediction of protein structures, functions and interactions using the IntFOLD7, MultiFOLD and ModFOLDdock servers. Nucleic Acids Res. 51, W274–W280. 10.1093/nar/gkad297 37102670 PMC10320135

[B32] Monticelli L. Kandasamy S. K. Periole X. Larson R. G. Tieleman D. P. Marrink S.-J. (2008). The MARTINI coarse-grained force field: extension to proteins. J. Chem. Theory Comput. 4, 819–834. 10.1021/ct700324x 26621095

[B33] Nguyen T. T. Tran D. P. Huy P. D. Q. Hoang Z. Carloni P. Van Pham P. (2016). Ligand binding to anti-cancer target CD44 investigated by molecular simulations. J. Mol. Model. 22, 165. 10.1007/s00894-016-3029-6 27342250

[B34] Nicolosi P. A. Dallatomasina A. Perris R. (2015). Theranostic impact of NG2/CSPG4 proteoglycan in cancer. Theranostics 5, 530–544. 10.7150/thno.10824 25767619 PMC4350014

[B35] Nishiyama A. Dahlin K. J. Prince J. T. Johnstone S. R. Stallcup W. B. (1991). The primary structure of NG2, a novel membrane-spanning proteoglycan. J. Cell Biol. 114, 359–371. 10.1083/jcb.114.2.359 1906475 PMC2289079

[B36] Nishiyama A. Lin X.-H. Giese N. Heldin C.-H. Stallcup W. B. (1996). Interaction between NG2 proteoglycan and PDGF α-receptor on O2A progenitor cells is required for optimal response to PDGF. J. Neurosci. Res. 43, 315–330. 10.1002/(SICI)1097-4547(19960201)43:3<315::AID-JNR6>3.0.CO;2-M 8714520

[B37] Nishiyama A. Stallcup W. B. (1993). Expression of NG2 proteoglycan causes retention of type VI collagen on the cell surface. Mol. Biol. Cell 4, 1097–1108. 10.1091/mbc.4.11.1097 8305732 PMC275746

[B38] Piovesan D. Del Conte A. Clementel D. Monzon A. M. Bevilacqua M. Aspromonte M. C. (2023). MobiDB: 10 years of intrinsically disordered proteins. Nucleic Acids Res. 51, D438–D444. 10.1093/nar/gkac1065 36416266 PMC9825420

[B39] Pluschke G. Vanek M. Evans A. Dittmar T. Schmid P. Itin P. (1996). Molecular cloning of a human melanoma-associated chondroitin sulfate proteoglycan. Proc. Natl. Acad. Sci. U. S. A. 93, 9710–9715. 10.1073/pnas.93.18.9710 8790396 PMC38494

[B40] Rivas G. Minton A. P. (2024). New developments in the study of biomolecular associations via sedimentation equilibrium. Trends biochem. Sci. 18, 284–287. 10.1016/0968-0004(93)90035-l 8236439

[B41] Rolih V. Barutello G. Iussich S. De Maria R. Quaglino E. Buracco P. (2017). CSPG4: a prototype oncoantigen for translational immunotherapy studies. J. Transl. Med. 15, 151. 10.1186/s12967-017-1250-4 28668095 PMC5494135

[B42] Sardone F. Santi S. Tagliavini F. Traina F. Merlini L. Squarzoni S. (2016). Collagen VI-NG2 axis in human tendon fibroblasts under conditions mimicking injury response. Matrix Biol. J. Int. Soc. Matrix Biol. 55, 90–105. 10.1016/j.matbio.2016.02.012 26944560

[B43] Schlessinger J. Plotnikov A. N. Ibrahimi O. A. Eliseenkova A. V. Yeh B. K. Yayon A. (2000). Crystal structure of a ternary FGF-FGFR-heparin complex reveals a dual role for heparin in FGFR binding and dimerization. Mol. Cell 6, 743–750. 10.1016/S1097-2765(00)00073-3 11030354

[B44] Soni J. Gupta S. Mandal T. (2024). Recalibration of MARTINI-3 parameters for improved interactions between peripheral proteins and lipid bilayers. J. Chem. Theory Comput. 20, 9673–9686. 10.1021/acs.jctc.4c00645 39491480

[B45] Stallcup W. B. (2002). The NG2 proteoglycan: past insights and future prospects. J. Neurocytol. 31, 423–435. 10.1023/A:1025731428581 14501214

[B46] Stallcup W. B. (2017). NG2 proteoglycan enhances brain tumor progression by promoting beta-1 integrin activation in both cis and trans orientations. Cancers 9, 31. 10.3390/cancers9040031 28362324 PMC5406706

[B47] Stallcup W. B. Dahlin K. Healy P. (1990). Interaction of the NG2 chondroitin sulfate proteoglycan with type VI collagen. J. Cell Biol. 111, 3177–3188. 10.1083/jcb.111.6.3177 2269670 PMC2116373

[B48] Staub E. Hinzmann B. Rosenthal A. (2002). A novel repeat in the melanoma-associated chondroitin sulfate proteoglycan defines a new protein family. FEBS Lett. 527, 114–118. 10.1016/s0014-5793(02)03195-2 12220645

[B49] Steentoft C. Vakhrushev S. Y. Joshi H. J. Kong Y. Vester-Christensen M. B. Schjoldager K. T.-B. G. (2013). Precision mapping of the human O-GalNAc glycoproteome through SimpleCell technology. EMBO J. 32, 1478–1488. 10.1038/emboj.2013.79 23584533 PMC3655468

[B50] Tamburini E. Dallatomasina A. Quartararo J. Cortelazzi B. Mangieri D. Lazzaretti M. (2019). Structural deciphering of the NG2/CSPG4 proteoglycan multifunctionality. FASEB J. 33, 3112–3128. 10.1096/fj.201801670R 30550356

[B51] Tan A. M. Zhang W. Levine J. M. (2005). NG2: a component of the glial scar that inhibits axon growth. J. Anat. 207, 717–725. 10.1111/j.1469-7580.2005.00452.x 16367799 PMC1571583

[B52] Tang F. Lord M. S. Stallcup W. B. Whitelock J. M. (2018). Cell surface chondroitin sulphate proteoglycan 4 (CSPG4) binds to the basement membrane heparan sulphate proteoglycan, perlecan, and is involved in cell adhesion. J. Biochem. (Tokyo) 163, 399–412. 10.1093/jb/mvy008 29462330 PMC5905647

[B53] Thomasen F. E. Skaalum T. Kumar A. Srinivasan S. Vanni S. Lindorff-Larsen K. (2024). Rescaling protein-protein interactions improves Martini 3 for flexible proteins in solution. Nat. Commun. 15, 6645. 10.1038/s41467-024-50647-9 39103332 PMC11300910

[B54] Tillet E. Gential B. Garrone R. Stallcup W. B. (2002). NG2 proteoglycan mediates beta1 integrin-independent cell adhesion and spreading on collagen VI. J. Cell. Biochem. 86, 726–736. 10.1002/jcb.10268 12210739

[B55] Tillet E. Ruggiero F. Nishiyama A. Stallcup W. B. (1997). The membrane-spanning proteoglycan NG2 binds to collagens V and VI through the central nonglobular domain of its core protein. J. Biol. Chem. 272, 10769–10776. 10.1074/jbc.272.16.10769 9099729

[B56] Touw W. G. Baakman C. Black J. te Beek T. A. H. Krieger E. Joosten R. P. (2015). A series of PDB-related databanks for everyday needs. Nucleic Acids Res. 43, D364–D368. 10.1093/nar/gku1028 25352545 PMC4383885

[B57] Tsirigos K. D. Peters C. Shu N. Käll L. Elofsson A. (2015). The TOPCONS web server for consensus prediction of membrane protein topology and signal peptides. Nucleic Acids Res. 43, W401–W407. 10.1093/nar/gkv485 25969446 PMC4489233

[B58] Ughrin Y. M. Chen Z. J. Levine J. M. (2003). Multiple regions of the NG2 proteoglycan inhibit neurite growth and induce growth cone collapse. J. Neurosci. Off. J. Soc. Neurosci. 23, 175–186. 10.1523/JNEUROSCI.23-01-00175.2003 PMC6742139 12514214

[B59] Wen Y. Makagiansar I. T. Fukushi J. Liu F.-T. Fukuda M. N. Stallcup W. B. (2006). Molecular basis of interaction between NG2 proteoglycan and galectin-3. J. Cell. Biochem. 98, 115–127. 10.1002/jcb.20768 16365873

[B60] Wong H. Crowet J.-M. Dauchez M. Ricard-Blum S. Baud S. Belloy N. (2022). Multiscale modelling of the extracellular matrix. Matrix Biol. Plus 13, 100096. 10.1016/j.mbplus.2021.100096 35072037 PMC8763633

[B61] Yu X. Martinez M. Gable A. L. Fuller J. C. Bruce N. J. Richter S. (2015). webSDA: a web server to simulate macromolecular diffusional association. Nucleic Acids Res. 43, W220–W224. 10.1093/nar/gkv335 25883142 PMC4489311

[B62] Zhang H. Wu Z. Hu D. Yan M. Sun J. Lai J. (2022). Immunotherapeutic targeting of NG2/CSPG4 in solid organ cancers. Vaccines 10, 1023. 10.3390/vaccines10071023 35891187 PMC9321363

[B63] Zhang S. Krieger J. M. Zhang Y. Kaya C. Kaynak B. Mikulska-Ruminska K. (2021). ProDy 2.0: increased scale and scope after 10 years of protein dynamics modelling with Python. Bioinformatics 37, 3657–3659. 10.1093/bioinformatics/btab187 33822884 PMC8545336

[B64] Zhen Y. Haugsten E. M. Singh S. K. Wesche J. (2018). Proximity labeling by a recombinant APEX2-FGF1 fusion protein reveals interaction of FGF1 with the proteoglycans CD44 and CSPG4. Biochemistry 57, 3807–3816. 10.1021/acs.biochem.8b00120 29812912

