# Corrigendum: Computational analysis of the structural-functional dynamics of a Co-receptor proteoglycan

**DOI:** 10.3389/fmolb.2025.1612818

**Published:** 2025-05-19

**Authors:** Francesco Tavanti, Giorgia Brancolini, Roberto Perris

**Affiliations:** ^1^ COMT – Centre for Molecular and Translational Oncology, University of Parma, Parma, Italy; ^2^ Department of Chemical and Life Sciences and Environmental Sustainability, University of Parma, Parma, Italy; ^3^ Center S3, CNR Institute of Nanoscience – CNR-NANO, Modena, Italy

**Keywords:** proteoglycans, molecular dynamics, computer simulations, co-receptor, growth factor

In the published article, there was an error in the **Funding** statement. The statement was incomplete. The correct **Funding** statement appears below.

