# Supplementary material for: Computational analysis of the structural-functional dynamics of a Co-receptor proteoglycan
Source: Front Mol Biosci. 2025 Mar 25;12:1549177. doi: 10.3389/fmolb.2025.1549177 (PMC11975855; doi:10.3389/fmolb.2025.1549177)
Supplement: Supplementary file 1 [file Supplementaryfile1.docx]

**Computational Analysis of the Structural-Functional Dynamics of a Co-receptor proteoglycan**

**Francesco Tavanti^1,2,3^*, Giorgia Brancolini^3^ and Roberto Perris^1,2^**

^1^COMT – Centre for Molecular and Translational Oncology

University of Parma

Italy

&

^2^Department of Chemical and Life Sciences and Environmental Sustainability

University of Parma

Italy

Email : [francesco.tavanti@unipr.it](mailto:francesco.tavanti@unipr.it)

^3^Center S3, CNR Institute of Nanoscience – CNR-NANO, Modena, Italy

*** Correspondence:**Corresponding Author

francesco.tavanti@unipr.it

The Martinize2 command line to build the CSPG4/NG2 model:

./martinize2 -o topol.top -x out.pdb -cys auto -elastic -ef 500.0 -el 0.5 -eu 1.0 -ea 0 -ep 0 -v -ss CCCCCCCCCCCCCCCCCSSCCSCCCCCCCEEEESSCCEEECCCSEESSEEEEEEEEECCSSEEEEEEECSSCEEEEEEETTEEEEEEEETTEEEEEECCTTCCSCSSSCEEEEEEEETTEEEEEETTTEEEEEECCSCCEECTTEEEESSCTTCCCGGGTTCCCBCEEEEEEEEETTEETTCSCCTTSEESCCGGGSCCTTSCEEECSTTCEEEESCCCCSSEEEEEEEEEECCSEEEEEEEEBSTTCCEEEEEEETTEEEEEEEETTEEEEEEEEEECTTSSEEEEEEEEESSEEEEEETTEEEEEECCSCCCCCCBCSCEEESSCCHHHHHHHHHTTCSCCHHHHTCCCCEEEEEEEETTEECCGGGCSEEESEEESCCCCCCCCCCCCCCCCCCCCCCCCCCCCCCCCSCCCCCCTTSCGGGTTCCCSEEECCEEEETTCEEECCGGGEEESSCTTTTTCCGGGEEEEEEECCSSEEEEECSTTHHHHTEEEHHHHHTTCEEEEECCCSCSEEEEEEEEEECCSSCCCHHHHSCEEEEEEEEEECCCCCCEEECTTCSEEEEETTCEEEECTTTCEEECTTSCGGGCEEEEESCSSCCCEEETTEEEEECSEEEHHHHHTTCEEEECCSSSEEEEEEEEECSSSBCCCEEEEEEEECCCCEEEEECCEEEETTCEEEECTTTEEEECCCSSCCCCEEEEECSCCSSEEEEEECSSTTTTTCEEECSEEEHHHHHTTCEEEEECCCSCCSSCEEEEEEEEEEETTEEEEEEEEEEEEECCCEEEEEECCEECCTTCCEEECTTTEEEEESSCCSSCCCCEEEEEECCSSEEEEETTEECCTTCEEEHHHHHTTCEEEEECCCCSSCEEEEEEEEEEETTEECCCEEEEEEECCCSCCCEEEECCEEEETTCEEEECTTTEEEECSSCCCEEEEECSCCSSEEEEESSCSCCCCCCSEEEHHHHHTTCEEEEECCCSCSEEEEEEEEEECCCCCCCCCCCEEEEEEEEEEECCCCCCCEEEEECCEEEETTCEEEECTTTEEEECCSTTCCGGGCEEEEECCSSCEEEETTEEEEECSEEEHHHHHTTCEEEECCSCSEEEEEEEEECSSSEEEEEEEEEEECCCEEECSCCCEEEETTCEEEECTTTSCEEESSCCCSTTSEEEEEEECCSSEEEEETTEECSEEEHHHHHTTCEEEEECCCSCSEEEEEEEEEETTEEEEEEEEEEEECCCCCCCCEEEEECCEEEETTCCEECCTTTEEEECTTCCGGGCEEEEEECCSSEEEEEEECCSSTTSCCEEEECSEEEHHHHHTTCEEEEECCTTCSEEEEEEEEECSSSCCEEEEEEEEEEECSSCCEEECCEEEETTCEEECCTTTEEECSSSGGGCTTEEEEEEECCSSEEEEETTCSSCCCCSEEEHHHHHTTCEEEEECCCSCSEEEEEEEEEETTTTEECCCEEEEEEEECCSCSCCEEEEECCEEEETTCEEECCTTTEEEECTTSCTTTCEEEECCCSSEEEEETTEEEEECSEEEHHHHHTTCEEEEECSCSEEEEEEEEECSSCBCCCEEEEEEEECCCCEEEESCSEEEECTTCEEECCTTTCEEEETTCCCGGGCEEEEEECCSSEEEEETTSCCCSCCCCEEEHHHHHTTCEEEEECCCSSCCSEEEEEEEEEEEETTEEEEEEEEEEEEESSSCCSSSCCSSSEECCEECCTTCEEEECTTTEECGGGGGGSCGGGGGGCEEEEEEEECCSSEEEEETTEECCTTSCEEEHHHHHTTCEEEEECCCSSCCEEEEEEEEEECTTSCEEEEEEEEEEEEEECCCCCCCCCEEECCCCEEEETTCEEECCTTTCEEECTTSCTTTCEEEEEECCTTCEEEETTSCSSCCSEEEHHHHHTTCEEEECCSCSSCEEEEEEEECSSSCCEEEEEEEEEECSCCEEECCSCEEEETTTCEEECCTTTCEEESSSBCTTCEEEEEECCSSEEEEETTEECSEEEHHHHHTTCEEEEESSTTCSEEEEEEEEEETTEEEEEEEEEEEECCCEECCCSCEEETCEEEECTTTEECHHHHHHHSSCCEEEEEECCTTEEEEECCSCSSCCCCCEECSEEEHHHHHTTCEEEEECCCSSCCCSCEEEEEEEEEECTTSCCEEEEEEEEEEECCTTSCCSSCEEECCCCCCCCCCCCCCCCCCCCCCCCCCCCCCCCCCCCCCCCSCCSCSSCSSSTTTSHHHHHHHHHHHHSSSCCCCCCCCC -p backbone -f prot.gro

**SUPPLEMENTARY FIGURES**


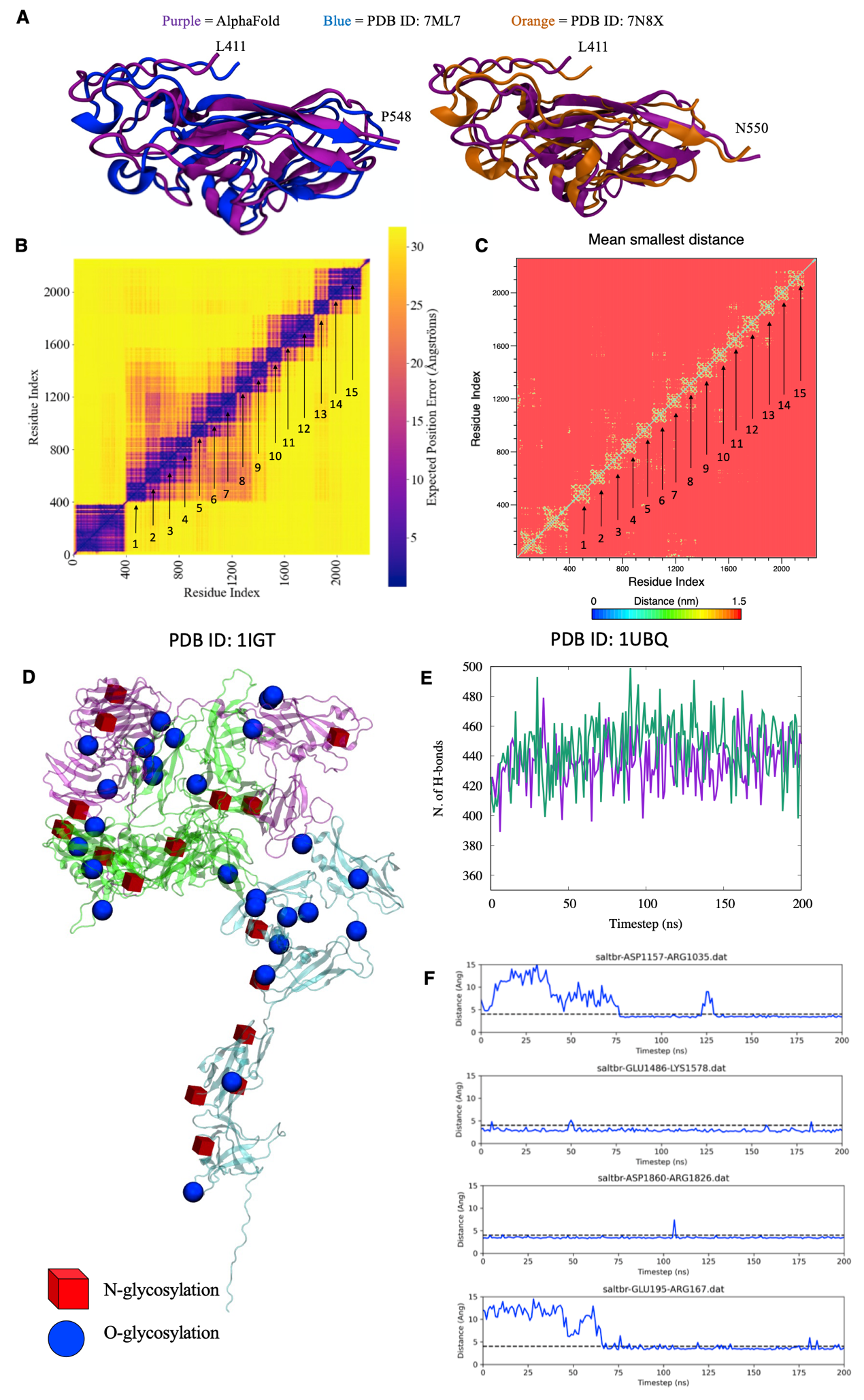


**Supplementary Figure 1**: A) comparison between experimental and AlphaFold models of the repeating unit 1. B) The PAE distribution obtained from AlphaFold with repeating units labeled with their number. C) the distribution of the minimum distance between amino acids with repeating units labeled with their number. D) representation of O- and N-glycosylation sites on the NG2/CSPG4 protein, rendered as blue spheres and red cubes respectively. E) Number of hydrogen bonds during the simulation time for the two atomistic replicas. F) the oxygen-nitrogen distance between amino-acids pairs that form a salt-bridge, considering a cutoff distance for bond formation of 4Å.


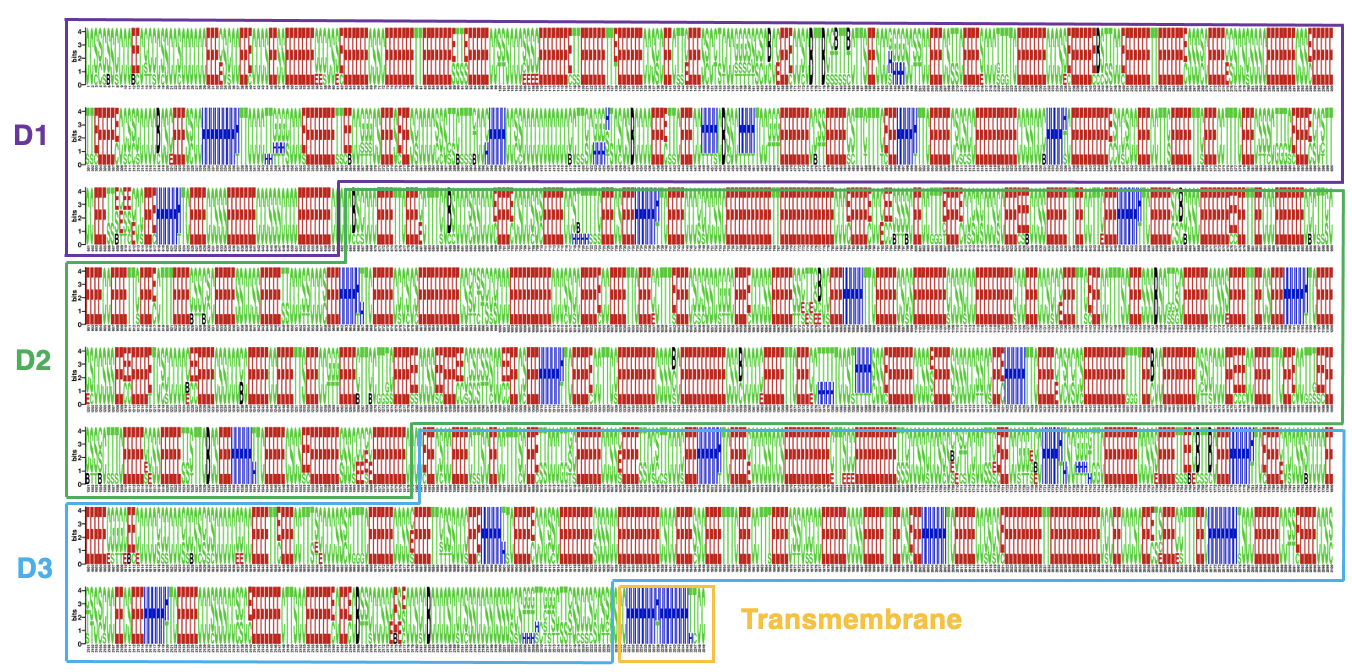


**Supplementary Figure 2**: Sequence logo of NG2/CSPG4 colored accordingly to the relative probability of secondary structure assignment for each amino acid: E = extended strands in red; H = alpha-helix in blue; T = hydrogen-bonded turns, S = bends and C = loops in green and B = residues in isolated beta-bridge in black.


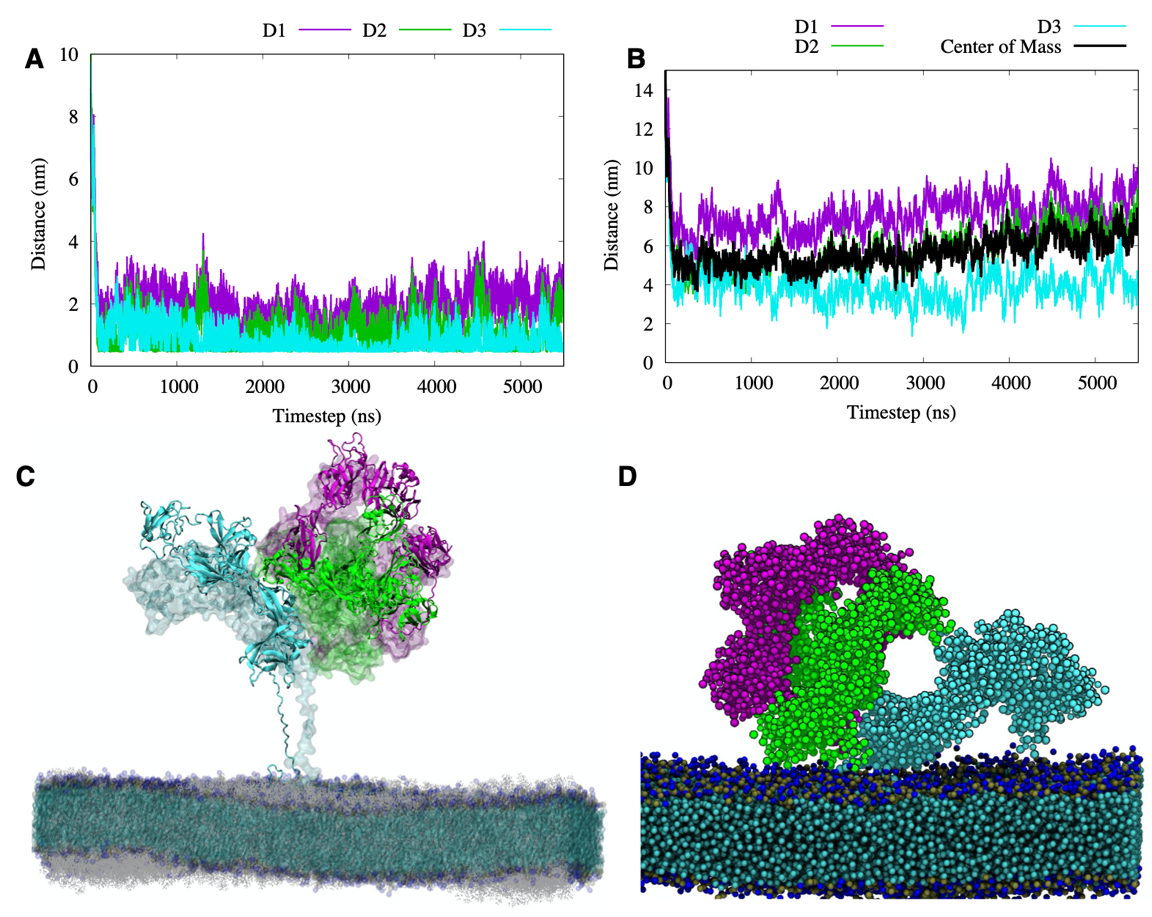


**Supplementary Figure 3: A)** and **B)** illustrate the minimum distance and the center of mass distances between each structural domain of NG2/CSPG4 and the membrane surface, as determined by the second replica of the CG simulation: the black line represents the Center of Mass of the three domains. **C)** comparison between the full-atomistic model and the CG one showing high degree of equivalence between the two models. **D)** illustrates the CG representation of the configuration of NG2/CSPG4 at 5.5µs.


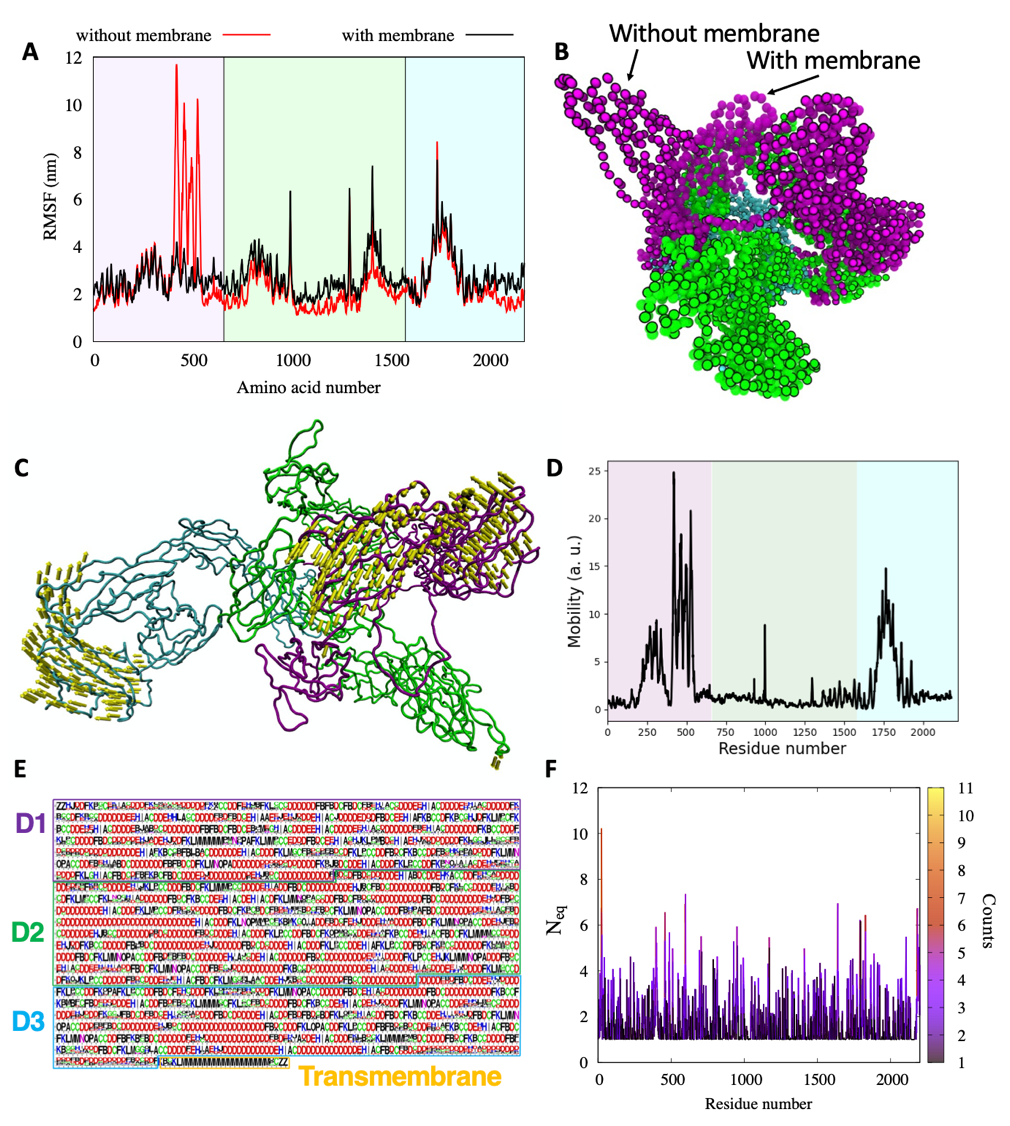


**Supplementary Figure 4**: **A**) and **B**) show the comparison between the RMSF and the configuration of the CG model with and without inclusion of the cell membrane. The higher RMSF values without the membrane are given by the D1 domain, which seems to be less in contact with the remainder of the protein. **C-D**) show the graphical representation and the mobility analysis of the first Normal Mode for NG2/CSPG4 with the adjacent cell membrane. ***Panel E***) reports the sequence logo plot of NG2/CSPG4 where each letter represents a different Protein Block as reported in (de Brevern *et al*, 2000). In **F**) the Number of equivalent structures (Neq) computed from the Protein Block analysis.


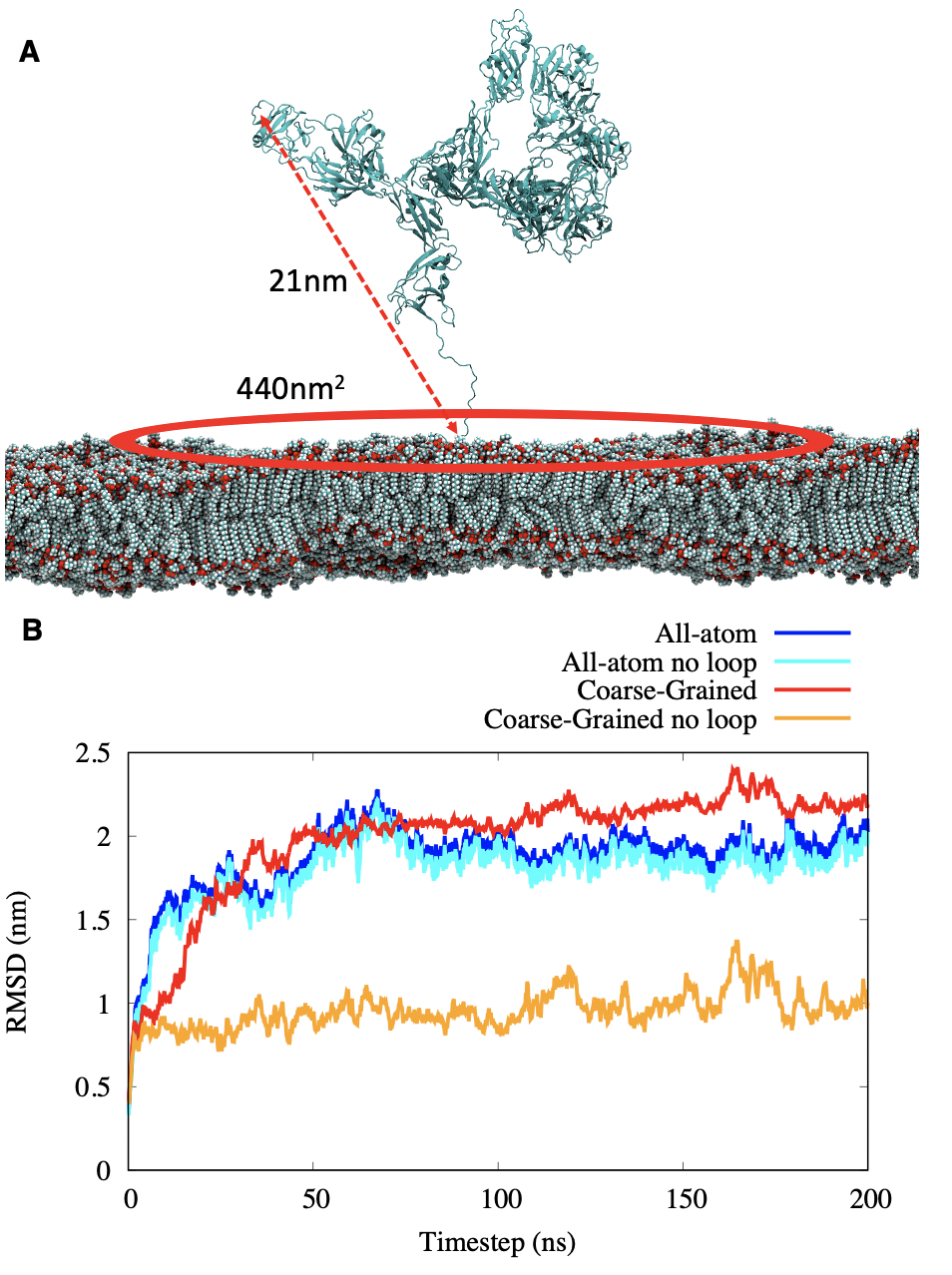


**Supplementary Figure 5**: **A**) Schematic representation of the possible interaction area of the NG2/CSPG4, considering the flexibility of the domain D3 tail. **B**) The RMSD calculation for the atomistic and Coarse-Grained model using all the protein (blue and red respectively) and only the residues 1 to 2180 of the protein core (cyan and orange respectively).


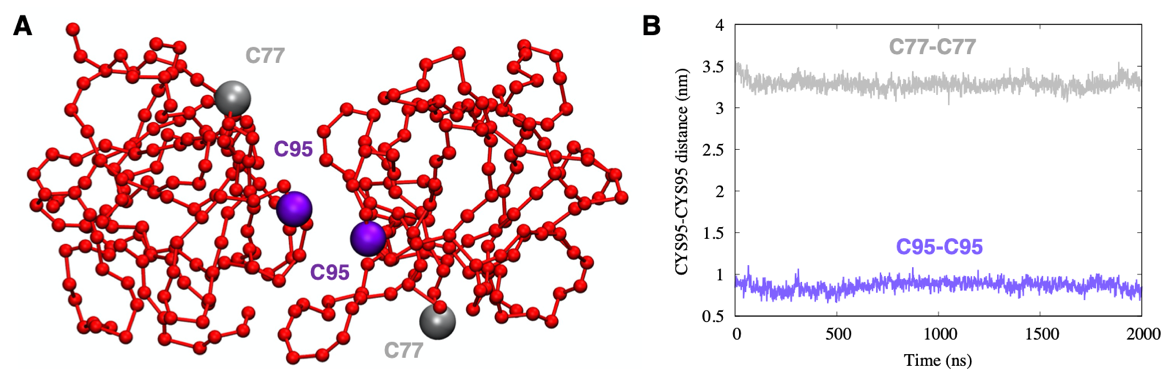


**Supplementary Figure 6**: **A**) spatial displacement of Cysteine residues (C77 and C95) involved in disulfide bonds, where red ball-and-sticks represent the FGF-dimer. In **B**) the distance between same Cysteine residues during the simulation time.

**Supplementary Table 1**: list of the N- and O-glycosylation sites expressed in terms of amino-acids number for the extracellular portion of NG2/CSPG4.

| N-glycosylation | 130, 348, 427, 685, 772, 1131, 1202, 1364, 1449, 1645, 1909, 2016, 2034,  2040, 2075 |
| --- | --- |
| O-glycosylation | 162, 174, 179, 399, 745, 794, 797, 803, 858, 954, 1134, 1144, 1169, 1301,  1307, 1489, 1596, 1601, 1608, 1639, 1739, 1824, 1847, 1960, 2102, 2184,  2191, 2195, 2203 |
